# On the functional characterization of lytic polysaccharide monooxygenases (LPMOs)

**DOI:** 10.1186/s13068-019-1392-0

**Published:** 2019-03-19

**Authors:** Vincent G. H. Eijsink, Dejan Petrovic, Zarah Forsberg, Sophanit Mekasha, Åsmund K. Røhr, Anikó Várnai, Bastien Bissaro, Gustav Vaaje-Kolstad

**Affiliations:** Faculty of Chemistry, Biotechnology and Food Science, Norwegian University of Life Sciences (NMBU), PO Box 5003, 1432 Ås, Norway

**Keywords:** LPMO, Biomass conversion, Cellulose, Chitin, Oxygen, Hydrogen peroxide

## Abstract

Lytic polysaccharide monooxygenases (LPMOs) are abundant in nature and best known for their role in the enzymatic conversion of recalcitrant polysaccharides such as chitin and cellulose. LPMO activity requires an oxygen co-substrate, which was originally thought to be O_2_, but which may also be H_2_O_2_. Functional characterization of LPMOs is not straightforward because typical reaction mixtures will promote side reactions, including auto-catalytic inactivation of the enzyme. For example, despite some recent progress, there is still limited insight into the kinetics of the LPMO reaction. Recent discoveries concerning the role of H_2_O_2_ in LPMO catalysis further complicate the picture. Here, we review commonly used methods for characterizing LPMOs, with focus on benefits and potential pitfalls, rather than on technical details. We conclude by pointing at a few key problems and potential misconceptions that should be taken into account when interpreting existing data and planning future experiments.

## Background

**Fig. 1:**
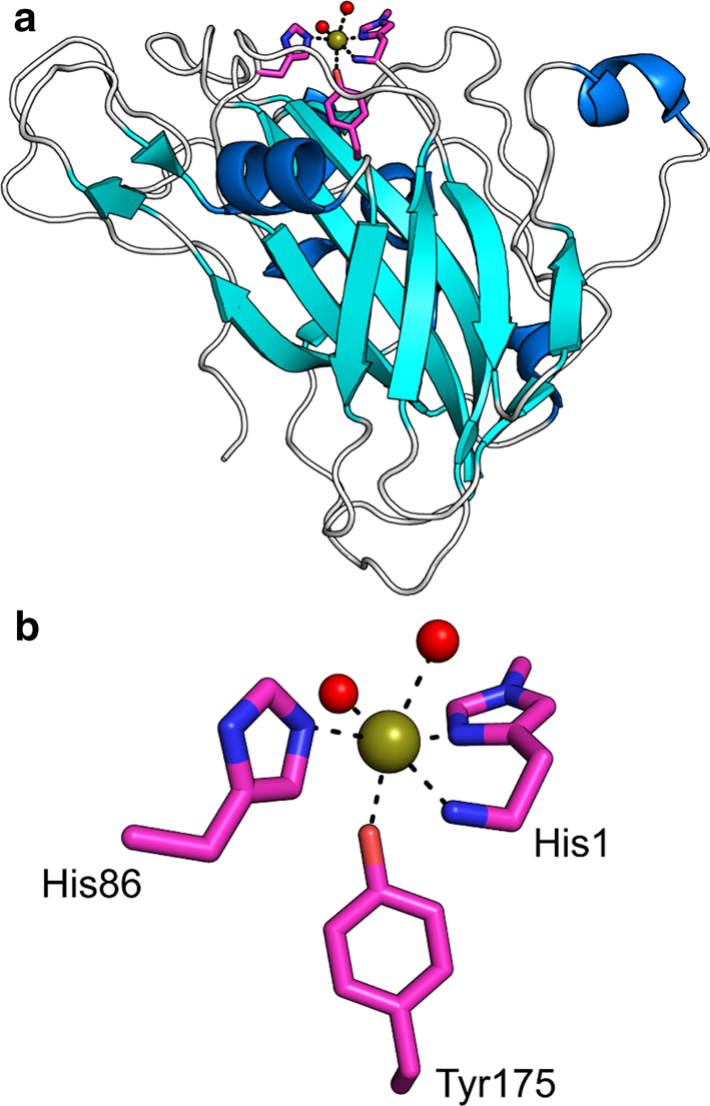
Three-dimensional structure of a typical LPMO and its active site. **a** The crystal structure and **b** details of the catalytic center of a cellulose-active family AA9 LPMO from the fungus *Thermoascus aurantiacus*, *Ta*LPMO9A (also known as *Ta*GH61A; [5], PDB ID: 2YET). The crystal structure is displayed in cartoon representation. The active site residues are shown as sticks with pink colored carbon atoms. The copper atom is shown as a golden sphere and water molecules coordinated by the copper atom are shown as red colored spheres. **b** A close up of the active site

**Fig. 2:**
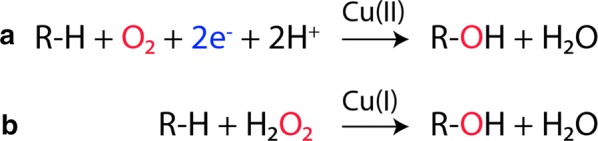
LPMO reaction schemes. The two panels show the reaction schemes for O_2_- and H_2_O_2_-driven LPMO activity proposed in **a** 2010 [3] and **b** 2017 [10]. The Cu(II)/Cu((I) indicated above the arrows refers to the copper ion in the active site and its oxidation state before initiation of the catalytic cycle. Note that in the O_2_-driven reaction, delivery of two electrons is needed for each catalytic cycle, whereas the H_2_O_2_-driven reaction only requires a “priming” reduction of the LPMO, which, once activated, can carry out multiple reactions

The discovery of lytic polysaccharide monooxygenases (LPMOs; Fig. 1) has profoundly changed the way in which we view the enzymatic conversion of polysaccharides, in particular recalcitrant materials such as chitin and cellulose. The boosting effect of LPMOs on the activity of classical hydrolytic enzymes was first described in 2005, for chitin [1] and in 2007, for cellulose [2]. In 2010, Vaaje-Kolstad et al. showed that these, at the time, enigmatic “boosting” proteins catalyze oxidative cleavage of glycosidic bonds, which suggested that LPMOs may be central players in a network of oxidoreductases involved in biomass conversion [3, 4]. LPMOs are mono copper enzymes [5, 6]. The copper is bound in a characteristic histidine-brace (Fig. 1), which is rare in Nature and which likely gives the LPMOs their remarkable oxidative power [5, 7, 8]. The LPMO reaction entails reduction of the copper by an external reductant, after which the enzyme reacts with either O_2_ [3, 9] or H_2_O_2_ [10–14] to form a powerful oxygen species that can hydroxylate the C1 or the C4 carbon in the scissile glycosidic bond [10, 15–17] (Fig. 2).

Characterization of LPMOs suffers from multiple complications, ranging from the production of active enzymes to characterizing their substrate specificity and kinetics. One particular issue, well known from work on other redox enzymes, but perhaps even worse for LPMOs, concerns the plethora of possible on- and off-pathway reactions that may take place when mixing reductants, O_2_ and/or H_2_O_2_, an insoluble, not necessarily “clean” substrate, the LPMO, and small amounts of free copper that may change during the reaction. As to the latter, progress curves for LPMO reactions are often non-linear, which in most cases is likely due to oxidative damage to the enzymes [10]. Such damage does not only lead to enzyme inactivation but also to release of copper in solution, even in otherwise “clean” experimental systems. To complicate things even further, LPMOs have oxidase activity, which implies that, in the presence of reductant, they may convert O_2_ to H_2_O_2_ [18, 19].

Because LPMOs are carbohydrate-active enzymes (CAZymes) they are classified in the CAZy database, which categorizes CAZymes on the basis of their sequence [20]. In the CAZy system, LPMOs are categorized as auxiliary activities (AA; [21]) and they currently make up six AA families: AA9, AA10, AA11, AA13, AA14 and AA15. The most widely studied LPMO families are AA9 and AA10.

Despite considerable progress in the LPMO field since 2010, functional characterization of these abundant and intriguing enzymes remains a major challenge. In this paper, we address the most common issues related to the production and characterization of LPMOs. We focus on practical aspects of characterizing functional properties, such as substrate specificity, reaction kinetics and stability, and pay particular attention to possible pitfalls. We also shortly discuss the possible importance of some of these pitfalls for interpreting recent studies on the nature of the LPMO co-substrate, O_2_ and/or H_2_O_2_. For details concerning the methodologies that we refer to, such as product analysis by mass spectrometry or liquid chromatography, or fundamental studies of copper-binding, we refer to recent research papers and reviews [6, 22–28].

### Production of active LPMOs

Most LPMOs characterized so far were recombinantly produced in *Escherichia coli*, for bacterial LPMOs, or the yeast *Pichia pastori*s, for fungal LPMOs, while a few were produced in fungal hosts. The fact that both the alpha-amino group and the side chain of the N-terminal histidine of the mature protein are involved in copper-binding (Fig. 1), and thus in catalysis, limits expression options. The most convenient way to produce enzymes with an N-terminal histidine is to export the proteins to the periplasmic space or culture medium, using appropriate signal peptides. Even when doing so, it is advisable to use proteomics technologies (i.e., fragmentation of the protein by trypsin and subsequent sequencing of the resulting peptides by mass spectrometry) to check that the signal peptide has been correctly processed and that the N-terminal residue indeed is a histidine, especially when using *Pichia* expression. LPMOs that become reduced in the absence of substrate and presence of O_2_ or H_2_O_2_ are prone to oxidative damage, especially the active site histidines (more details below). This is another reason for checking the recombinantly produced proteins using proteomics techniques; see [29] for an example. Of note, it is possible that a mixture of correctly and incorrectly processed LPMOs, with and without oxidative damage, appears as a homogeneous band on an SDS-PAGE gel, which hides the protein’s physical (and functional) heterogeneity.

Heterologous expression of LPMOs creates some challenges. Glycosylation may occur in the linker regions of certain actinomycete multi-domain proteins [30, 31] and will be absent when expressing such proteins in *E. coli*. Most fungal enzymes will be glycosylated and while glycosylation will also occur during expression in *P. pastoris*, the glycosylation patterns will usually be different compared to the natural host. The N-terminal histidine of fungal LPMOs carries a methylation [5] and this post-translational modification will not occur when these enzymes are produced in *P. pastoris*, as shown by the crystal structures of *Pichia*-produced LPMOs (e.g., [32–34]) and analysis of the N-terminal peptide of *Pichia*-produced LPMOs using proteomics technologies [35]. Petrovic et al. have recently shown that many functional properties of a family AA9 LPMO from the thermophilic fungus *Thermoascus aurantiacus, Ta*LPMO9A, including substrate specificity, redox potential, copper-binding and the ability to activate O_2_, are not affected by methylation of the N-terminal histidine [35]. The only difference found when comparing methylated *Ta*LPMO9A, produced in *Aspergillus*, with non-methylated *Ta*LPMO9A, produced in *P. pastoris*, was that the non-methylated form showed a lower operational stability (i.e., a higher degree of enzyme inactivation during reactions) and thus likely has a lower resistance against oxidative damage. Of note, the two enzyme forms had slightly different glycosylation patterns [35], and it cannot be excluded that this explains part of the observed differences in operational enzyme stability [35]. Several fungal LPMOs described in the current literature have been expressed in *P. pastoris* and these enzymes are active. While currently available data indicate that the N-terminal histidines of *Pichia*-produced LPMOs are not methylated, it must be noted that the methylation status of several *Pichia*-produced LPMOs appearing in the literature has not been analyzed.

Considering the importance of both the N-terminal amino group and the side chain of His 1 (Fig. 1b), the use of N-terminal purification tags is not possible when the goal is to produce active LPMOs, unless one has an efficient way to remove the tag after purification precisely in front of what needs to become the N-terminal histidine. C-terminal purification tags may sometimes be acceptable although, on a general note, we discourage use of tags since they may affect binding to the complex co-polymeric substrates of LPMOs. C-terminal His-tags have been successfully used and yielded active LPMOs [36, 37], however, we have experienced that the use of this tag may create complications in analysis of the enzyme due to its affinity for metal ions, including copper. LPMOs are secreted and tend to be stable and well-behaved proteins; their purification using standard chromatographic techniques that are not based on tags, such as ion exchange, hydrophobic interaction and size exclusion chromatography, tends to be rather straightforward. Reported storage temperatures for LPMOs are 4, − 20 and − 80 °C, but so far no studies have investigated the effect of storage temperature on enzyme stability.

LPMOs need copper to be active. Due to the high affinity for copper, with *K*_d_ values in the order of 1 nM for Cu(I) and 50 nM for Cu(II) [5, 6, 38], purified LPMOs will usually contain copper or pick up copper when incubated with substrates that contain this metal ion. To ensure full copper saturation, several approaches are possible. Direct addition of Cu(II) ions to reaction mixtures is not usually a good idea since a surplus of this transition metal in a reaction solution that also contains a reductant and O_2_ or H_2_O_2_, will promote a variety of side reactions. An approach commonly used entails incubation of the LPMO with a 1.5–3-fold molar surplus of Cu(II) ions, followed by removal of excess copper by size exclusion chromatography [27, 39]. Such procedure is often used as the final step in an LPMO purification strategy. Of note Cu(II) solutions should be made in pure water and kept at slightly acidic pH (around 3–4) since copper may precipitate as Cu(OH)_2_ in neutral or alkaline solutions.

If one intends to estimate the copper-binding affinity of the LPMO, divalent metal ions can be removed from the protein (and buffer) using EDTA. All buffers used downstream of the EDTA treatment must be metal free, which can be achieved through treatment with, e.g., the Chelex 100 resin [27, 40]. EDTA is an efficient divalent metal chelator, with an association constant of 10^18.78^ M^−1^ for Cu(II) [41]. Removal of Cu(II) from the LPMO active site is performed by playing on the LPMO-Cu(II) ↔ apo-LPMO + Cu(II) equilibrium (*K*_d_ ~ 50 nM; [6, 7, 40, 42]) by incubating the LPMO-Cu(II) solution with an excess of EDTA for a sufficient amount of time. Note that the lower the pH the less efficient EDTA might be as Cu(II) chelator due to partial protonation of carboxylic functions. In practice, in our lab, we incubate the LPMO-Cu(II) solution with 10 mM EDTA, at pH ~ 6, overnight, at 4 °C.

The proportion of copper atoms per molecule of LPMO may be assessed using EPR or ICP-MS [27]. However, not every lab may have easy access to such equipment and/or have the required expertise for routine controls. As an alternative, fluorescence measurements may be used, since measuring fluorescence is fast and usually requires low amounts of protein, while fluorimeters are widely accessible. The coordination of copper by an LPMO quenches its intrinsic fluorescence signal [38, 43], to an extent that is dependent on the copper redox state, Cu(II) being a stronger quencher than Cu(I) [43]. The magnitude of the effect varies, however, from LPMO to LPMO. We have noticed that AA10s usually provide a better response than AA9s. In practice, one can compare the fluorescence signal of an apo-enzyme vs a copper-saturated enzyme. Whether or not a transition from the Cu(II) to the Cu(I) state can be observed (i.e., an increase in fluorescence) may be assessed by looking at the effect of adding stoichiometric amounts of a good reductant (e.g., ascorbic acid) [43]. A properly prepared apo-LPMO should not show any increase in fluorescence. Another alternative is to measure UV–Vis absorbance, but this requires much higher amounts of enzyme.

### Basic characterization of LPMO activity using polysaccharide substrates

There are numerous ways to assess LPMO activity. The most relevant and informative methods entail incubation with a reductant and substrate followed by analysis of soluble products (i.e., oxidized oligosaccharides) by MALDI-TOF mass spectrometry (MS), which is fast and simple, or high performance liquid chromatography (HPLC), which is slightly more demanding. Importantly, control reactions without added reductant should always be performed, since LPMO preparations may be contaminated with regular glycoside hydrolases such as cellulases. Even trace amounts of such contaminating enzymes may have a profound effect on the product profile, in particular because LPMO reactions are relatively slow (see below). In reactions without added reductant, the LPMO will not be active, meaning that contaminating background activities can be detected. Since LPMO substrates may contain some reducing power, control reactions without added reductant may not always completely abolish LPMO activity, and oxidized products may still be detected. In such cases, sometimes, one may wish to do additional control experiments, for example, using EDTA to abolish LPMO activity.

Both LPMO activity and stability are affected by the type and concentration of the reductant and reductant properties depend on pH [44–46], as discussed below. The overarching impression from almost 10 years of LPMO research is that ascorbic acid generally gives good results in a relatively broad pH range. In a typical “first test” of LPMO activity one would use 1 mM ascorbic acid as reductant at a pH near 6. The choice of substrate obviously is of major importance, as discussed in detail below. Easily accessible substrates for initial testing include Avicel, phosphoric acid-swollen cellulose (PASC) prepared from Avicel [47] and commercially available α-chitin. Chitin-active LPMOs tend to be most active on β-chitin which is available for purchase through companies such as France Chitine (Orange, France) or that can be purified from squid pens using a relatively simple purification procedure (see [48] and references therein).

Although MS analysis of products sometimes can give a quantitative impression of enzyme activity, MS is primarily a qualitative method, providing a fast and simple way to assess activity and substrate specificity (the latter is discussed in more detail below). The masses of C1- and C4-oxidized products are identical, but it may still be possible to derive information about oxidative regioselectivity, as discussed in detail by Westereng et al. in [25, 28]. Oxidation at C4 yields a 4-keto-sugar that is in equilibrium with a gemdiol form (i.e., a hydrated 4-keto-sugar). These two variants of the oxidized species will usually appear as single sodium adducts. On the other hand, the lactone produced by C1-oxidation is in equilibrium with an aldonic acid form (i.e., a carboxylic group), which dominates at neutral pH. This aldonic acid form yields characteristic and often dominant MS signals due to the formation of salts, usually sodium salts. These “sodium salts of sodium adducts” have characteristic masses due to the presence of two sodium ions. The absence of such salt signals in spectra that show oxidized species strongly indicates that oxidation happens at C4. There are characteristic MS signals for products that are oxidized in both ends and, while these signals usually are small, they do appear when analyzing products of LPMOs that can act on both C1 and C4 (see, e.g., Fig. S1 in the study by Forsberg et al. [40]).

It is important to note that the most abundant cations that form adducts with LPMO products are sodium (Na^+^, 22.9897 Da) and potassium (K^+^, 39.0983 Da). The atomic masses of these elements differ from each other by approximately the atomic mass of oxygen (O, 15.9994 Da) and this may create problems. For example, the potassium adduct of a native oligosaccharide (M + 39) will have the same mass as the sodium adduct of a corresponding oxidized (M-2) and hydrated (M + 18) oligosaccharide (M-2 + 18 + 23). To avoid these complications, saturation with LiCl may be performed, leading to lithium (Li^+^, 6.941 Da) adducts only. To avoid false interpretation of results, the saturation level must be ensured, since the difference between the atomic masses of Li and Na is also approx. 16 Da. It should be noted that the above considerations are based on the use of MALDI-TOF MS, which is readily accessible in most laboratories. An alternative would be to use other types of mass spectrometers (e.g., Orbitraps) that offer a resolution that is so high that the nature of the adduct may be inferred solely from the measured mass of the analyte.

Standard HPLC methods for the separation of oxidized chito-oligosaccharides (C1-oxidized only), based on hydrophilic interaction chromatography (HILIC) with UV-detection, and oxidized cello-oligomers (C1, C4, and double oxidized C1/C4), based on high performance anion exchange chromatography with pulsed amperometric detection (HPAEC-PAD), are very well developed, giving baseline separation of all native and C1-oxidized soluble LPMO products ([3, 15, 22], see Vu et al. [49] for nice examples for cellulose). C4-oxidized products, which hitherto have only been observed for glucan substrates, are unstable at the alkaline conditions used in the chromatography, but do give reasonably well separated characteristic peaks that provide information on product length [24]. Importantly, under alkaline conditions, C4-oxidized products are converted to native oligomers [24], which explains the seemingly high production of native products by C4-oxidizing LPMOs. A second reason why native oligosaccharides may be found in LPMO reactions is the presence of hydrolase contaminants in the LPMO enzyme batch, as discussed above. Of note, the stability of C4-oxidized products is likely affected by temperature, so it is important to be aware of how one chooses to stop reactions; boiling has been used [50], but may not always be the best solution. Filtration, to separate the enzyme from the insoluble substrate, provides an alternative.

HPLC methods similar to those developed for analyzing native and oxidized cello-oligomers can also be used to detect LPMO products derived from xyloglucan, glucomannan, and mixed-linkage glucan [51, 52] and xylan [53, 54]. While chromatographic analysis will easily reveal LPMO activity on hemicellulosic substrates, detailed interpretation of product profiles is challenging because: (i) in contrast to cellulose, hemicellulosic polysaccharides and longer oligosaccharides are often water-soluble and hence the reactions yield complex product mixtures and chromatograms (as compared to chromatograms showing the limited set of soluble oligomeric products that may emerge in reactions with cellulose), and (ii) hemicellulosic oligosaccharides have diverse structures and pure standards are usually not available. Chromatographic profiles can be partially simplified by trying to reach reaction end-points, i.e., the point when all substrate has been converted to the shortest possible products. Alternatively, hydrolysis of the LPMO products with one or more suitable GHs may give simpler chromatograms (e.g., [55]; see also below).

Product quantification requires standards and a simplification of the product mixtures. The latter can be achieved by treating the products with glycoside hydrolases that convert oligomeric LPMO products to mixtures of oxidized mono-, di- and trimers, depending on the type of substrate and the enzymes used. Qualitative and quantitative C1-oxidized cello-oligosaccharide standards can be enzymatically produced using cellobiose dehydrogenase (CDH), which oxidizes cellobiose and longer cello-oligosaccharides [56, 57] to their corresponding aldonic acids (GlcGlc1A–Glc_n_Glc1A), as in refs. [52, 58]. A β-glucosidase may be used to convert C1-oxidized products to glucose and gluconic acid (Glc1A), where the latter is commercially available and can be used as a standard for oxidized products [59]. Of note, β-glucosidases cannot degrade cello-oligosaccharides that have been oxidized at the C4 position.

Degradation reactions with modern cellulase cocktails containing multiple LPMOs and cellulases will usually yield two oxidized products, gluconic acid and C4-oxidized cellobiose (Glc4gemGlc) [50, 59, 60]. A C4-oxidized dimer standard has been produced using LPMO9C from *Neurospora crassa* [19] to degrade cellopentaose to equimolar amounts of Glc4gemGlc and cellotriose, which allows indirect quantification of Glc4gemGlc by quantifying the amount of cellotriose [60]. While very useful, this latter quantification method needs to be used with great care, since, as discussed above, the C4-oxidized products are unstable and suffer from on-column degradation of the oxidized products during HPAEC-PAD [24, 50]. It is thus very important that the standard and the samples are treated in exactly the same way (exposure to pH, temperature, etc.).

Standards of oxidized chito-oligosaccharides with a degree of polymerization of 1–6 have been prepared using an AA7 chito-oligosaccharide oxidase from the fungal pathogen *Fusarium graminearum* (*Fg*ChitO; [61]) [39]. Standards for products derived from other common LPMO substrates, such as xyloglucan, are not available.

If one has access to powerful, LPMO-free cocktails of appropriate glycoside hydrolases, it is also possible to determine the total amount of LPMO-catalyzed cleavages rather than only determining oxidized soluble products [62–64]. In this case, subsequent to the LPMO reaction, all material in the reaction tube is converted to short oligomers, including oxidized short oligomers that elute with distinct retention times during HPLC. It is important to note that the ratio between soluble and insoluble oxidized sites will depend on the reaction set-up and will vary during the reaction. In an experiment using regenerated amorphous cellulose as substrate, Frommhagen et al. showed that the insoluble substrate fraction contained the majority of oxidized sites early on in the reaction and that the degree of solubilization of oxidized sites increased over time [63]. Loose et al. observed similar results for chitin-active LPMO variants with low activity [65]. In experiments with Avicel [62], Courtade et al. showed that the fraction of solubilized oxidized sites depends on the substrate concentration: the higher this concentration, the larger the fraction of oxidized sites in the insoluble substrate. Clearly, analysis of only the soluble fraction in LPMO reactions leads to various degrees of underestimation of LPMO activity.

To increase the general quality of activity assays, it is worth paying some attention to the preparation of reagents. One important aspect is to minimize the occurrence of trace metals that could promote auto-oxidation of the reductant and generation of reactive oxygen species. Reductants such as ascorbic acid should preferably be prepared in “trace select” water (Merck) and stock solutions need to be aliquoted and frozen at − 20 °C. Optimally, reductant solutions should be freshly made for each experiment. We recommend flushing the “trace select” water with nitrogen gas prior to dissolving the reductant. If one is to use H_2_O_2_, stock dilutions should be made in “trace select” water, in the dark, and the solutions should be aliquoted and stored at − 20 °C. It is important to check the H_2_O_2_ concentration experimentally and not just rely on information given on the bottle label.

### Side reactions

Even the most meticulously designed activity assays will suffer from multiple complications that need consideration when interpreting experimental data, depending on the purpose of the study. These complications derive from the fact that side reactions are almost unavoidable, especially when using complicated substrates that may contain reducing compounds or small amounts of transition metals. Things to consider include:

- The reductant may react with O_2_ and/or with H_2_O_2_, if the latter accumulates in the reaction mixture. Reactions between the reductant and O_2_ may generate H_2_O_2_. The extent of these reactions depends on the reductant (see, e.g., [46]).
- Reduced LPMOs that are not bound to substrate will, under aerobic conditions, produce H_2_O_2_ [18].
- Reduced LPMOs are prone to oxidative (self-)inactivation, regardless of whether the LPMO reaction is driven by O_2_ [64, 65] or H_2_O_2_ [10, 12]. Substrate-binding (i.e., high substrate concentration) protects against inactivation; substrate concentrations may significantly change during certain experimental set-ups (e.g., in applied bioprocessing type of studies), and so may LPMO stability.
- Although there may be debate on the nature of the true co-substrate of LPMOs, there is no doubt that H_2_O_2_ may drive the catalytic reaction for several LPMOs [10–12, 66]. So, at least for some LPMOs, varying levels of H_2_O_2_ in reaction mixtures may affect LPMO activity.
- H_2_O_2_ may engage in processes that can damage any enzyme in the reaction mixture, e.g., through Fenton chemistry-type of reactions [67].
- Several of the complicating processes listed above will be affected by the presence of transition metals. The concentration of transition metals may be affected by the type of substrate, the age of the substrate suspension, the degree of degradation of the substrate (which may affect metal release in solution), and LPMO inactivation (which will lead to release of copper in solution).
- The concentration of dissolved O_2_ is temperature dependent [e.g., ca. 8.3 mg/L (260 μM) at 25 °C and 5.6 mg/L (175 μM) at 50 °C, at atmospheric pressure, in fresh water; 68].

Several of these complexities are discussed in more detail, below.

### Fueling LPMO reactions with H_2_O_2_

Figure 2 shows reaction schemes for O_2_- and H_2_O_2_-driven LPMO reactions. The O_2_-driven reaction requires amounts of reductant that are stoichiometric relative to the amount of products formed, whereas the H_2_O_2_-driven reaction only requires priming amounts of reductant. In the latter scenario, a reductant will still be needed during the course of a reaction because LPMOs will occasionally be re-oxidized (see [13] for an in-depth analysis).

**Fig. 3:**
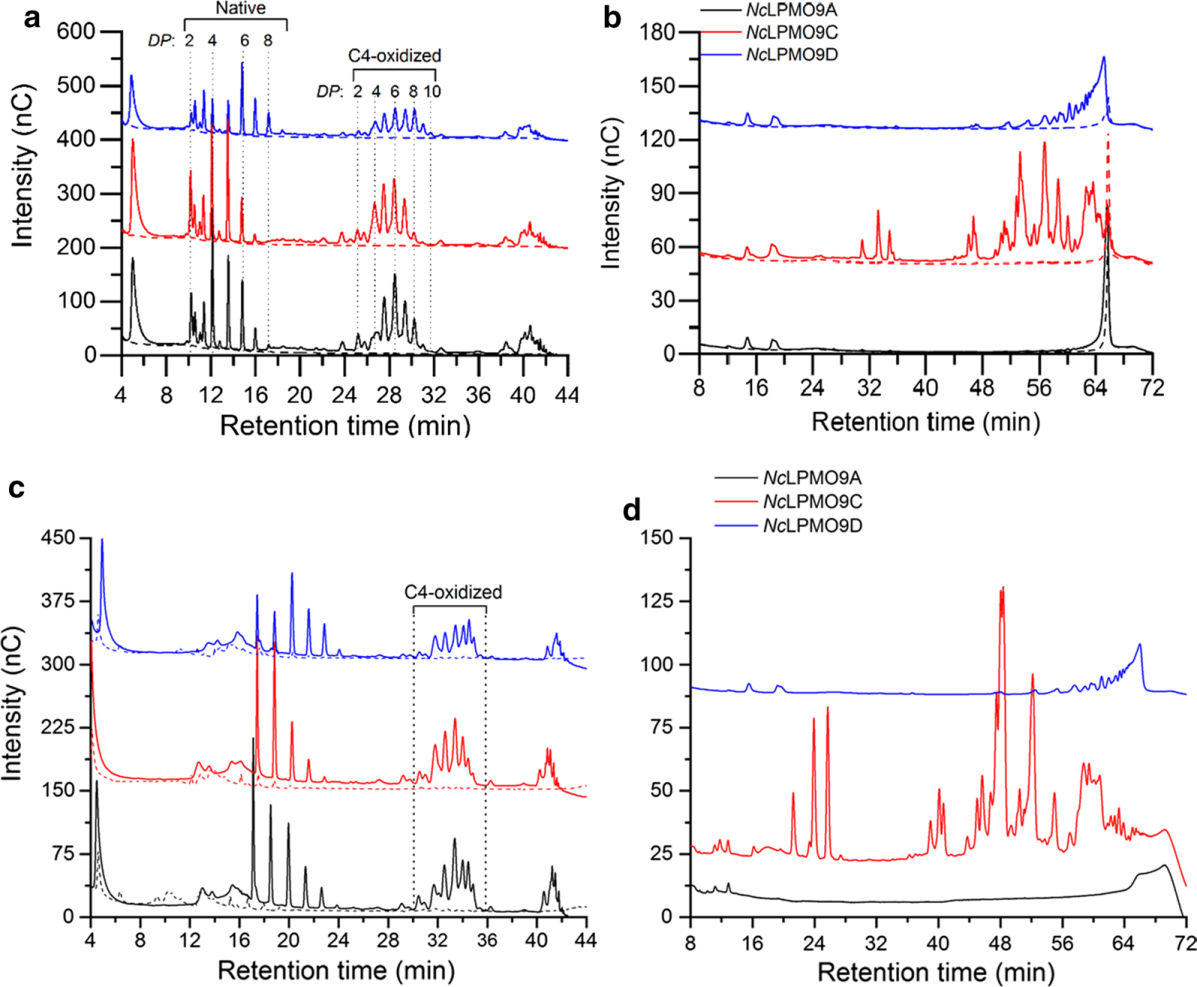
Soluble products generated by C4-oxidizing *Nc*LPMOs from PASC or TXG in reactions fueled by O_2_/Ascorbic acid or H_2_O_2_. **a**, **b** HPAEC-PAD profiles of products generated in reaction mixtures containing 1 mM ascorbic acid and 1 μM *Nc*LPMO9A (black line), 1 μM *Nc*LPMO9C (red line) or 1 μM *Nc*LPMO9D (blue line) and 2 mg mL^−1^ of **a** PASC or **b** TXG. **c**, **d** HPAEC-PAD profiles of products generated in reaction mixtures fueled by H_2_O_2_ containing 1 μM *Nc*LPMO9A (black line), 1 μM *Nc*LPMO9C (red line) or 1 μM *Nc*LPMO9D (blue line), and 2 mg mL^−1^ of **c** PASC or **d** TXG. In these latter reactions, ~ 45 μM of H_2_O_2_ was added to the reactions every 15 min; prior to every addition of H_2_O_2_, ~ 12 μM of ascorbic acid was added to ensure reduction of the LPMO. All the reactions were performed in standard aerobic conditions, i.e., in the presence of approximately 250 μM O_2_. The labeling of cello-oligosaccharides in **a** and **c** is based on previous work [19]. The large variation in retention times between **a** and **c** and between **b** and **d** is due to the fact that chromatograms were produced at different time points; in between, both columns and parts of the chromatographic system were replaced. These figures are derived from an unpublished study by Petrovic et al., which will be published elsewhere

There is some controversy in the field as to the nature of the natural oxygen co-substrate of LPMOs, O_2_ or H_2_O_2_. Regardless, it is now well documented, by several laboratories, using various LPMOs (AA9, AA10, AA11) and various substrates, that LPMOs can use H_2_O_2_ as a co-substrate and that H_2_O_2_-driven reactions are faster than O_2_-driven reactions [10–13, 35, 50, 66, 69]. It has been claimed that H_2_O_2_-driven reactions are less specific than O_2_-driven reactions and lead to products with atypical oxidation patterns [11]. In our experience, working with multiple LPMOs, from different families, with different oxidative regio-selectivities and with different substrates, there is no reduction of enzyme specificity when using H_2_O_2_ (Fig. 3). We cannot exclude that minor amounts of aspecifically oxidized products are generated in certain reactions, for example because an LPMO that is becoming oxidatively damaged slowly becomes less specific, as suggested by Hangasky et al. [11]. It is also possible that a suboptimal LPMO-substrate combination leads to a disturbed active site configuration in the enzyme–substrate complex that no longer precisely directs the reactive oxygen species to its correct destination, as suggested by results described by Simmons et al. [70]. It is not obvious, however, that the extent of these non-specific processes depends on the nature of the co-substrate, as discussed in more detail below.

**Fig. 4:**
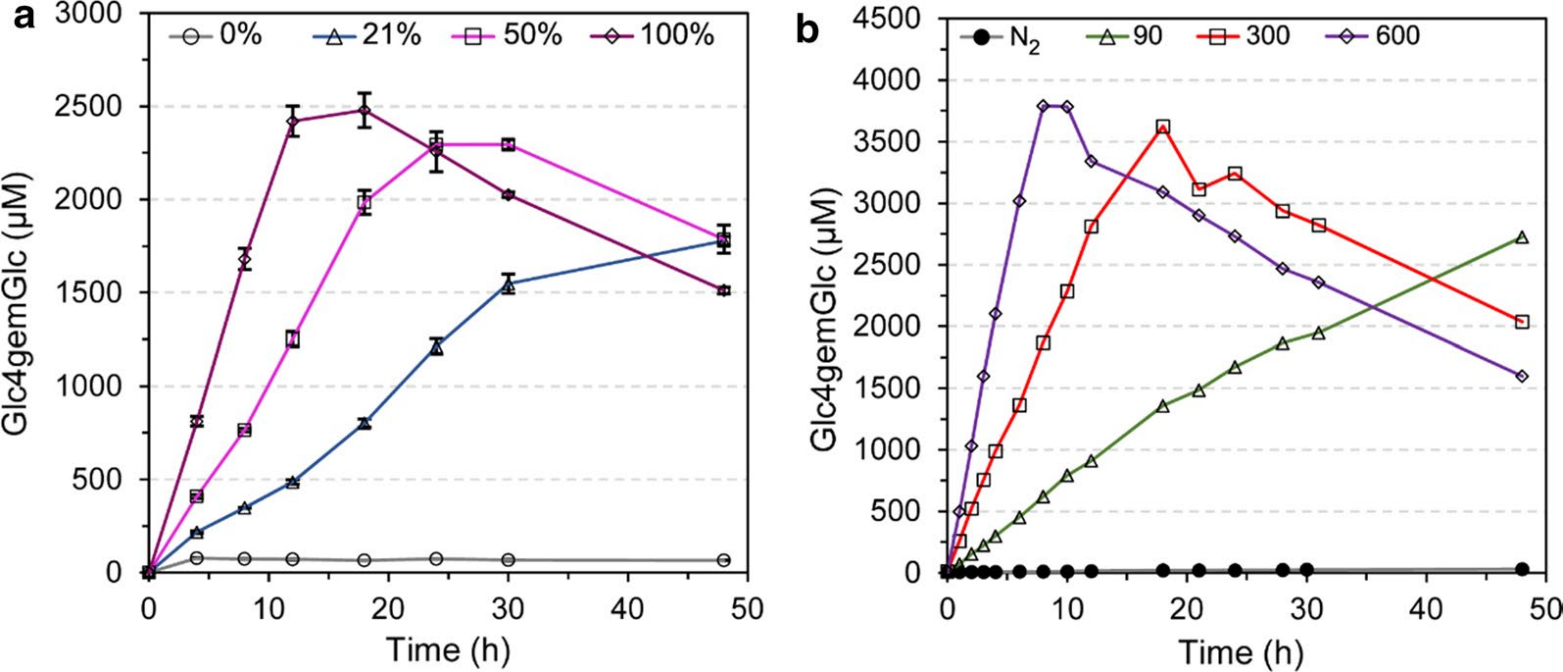
Inactivation of LPMOs. The graphs show formation of C4-oxidized cellobiose, the by far dominant soluble oxidized product, during degradation of Avicel with the commercial cellulase cocktail Cellic CTec2. **a** Product formation in reactions containing 5 mM ascorbic acid and varying oxygen concentrations, showing that higher oxygen concentrations give higher rates and faster inactivation of LPMOs. **b** Product formation in anaerobic reactions containing 1 mM ascorbic acid, with feeding of H_2_O_2_. The feeding rate of H_2_O_2_ in μM/h is indicated in the Figure. Increasing amounts of H_2_O_2_ give faster rates and faster inactivation of the enzyme. The gradual decrease in product levels is due to the instability of the product. This Figure was adapted from [50]

**Fig. 5:**
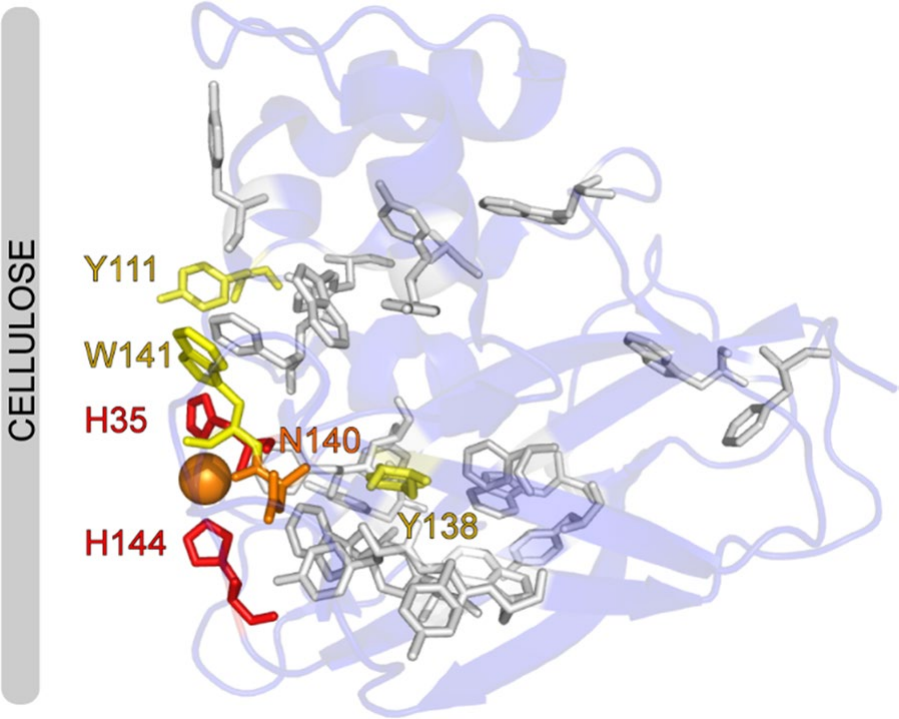
Oxidative damage of *Sc*LPMO10C (CelS2). Analysis of protein oxidation by proteomics techniques has demonstrated that a family AA10 LPMO from the actinobacterium *Streptomyces coelicolor*, *Sc*LPMO10C, exposed to protein inactivating conditions (presence of a reducing agent, but no substrate) is oxidized in and near the active site, predominantly on the catalytic histidines H35 (at the N terminus) and H144. The color code highlights the degree of oxidation: high (red), middle (orange) and low (yellow). For aromatic residues shown as gray sticks, no modification was detected. The grey cellulose fibril indicates the side of the protein where substrate will bind. The copper ion is shown as an orange sphere. The PDB code for *Sc*LPMO10C is 4OY7. The Figure was adapted from [10]

LPMOs are prone to autocatalytic oxidative inactivation in both O_2_-driven and H_2_O_2_-driven reactions [10, 50, 64, 65] (Figs. 4, 5) and the degree of inactivation will depend on the substrate type and concentration, as discussed below. The key problem when setting up LPMO reactions with added H_2_O_2_ is to avoid LPMO inactivation. Kinetic studies of a chitin-active LPMO indicate that the potentially detrimental reaction of a non-substrate bound reduced LPMO with H_2_O_2_ is up to three orders of magnitude slower than the productive reaction with substrate [12]. Still, at H_2_O_2_ concentrations that are high relative to the amount of LPMO and the amount of substrate, detrimental reactions in solution will occur, leading to inactivation of the LPMO. Depending on the type of reaction, overfeeding with H_2_O_2_, i.e., feeding with amounts of H_2_O_2_ that are higher than what the LPMOs can handle in a productive manner, may have additional negative consequences: the reductant may become depleted due to oxidation by H_2_O_2_, and/or the H_2_O_2_ may engage in other detrimental processes described above, in the “Side reactions” section.

It is important to note that the rates obtained in reactions with H_2_O_2_, both for productive catalysis and enzyme inactivation, may be orders of magnitude higher than what one is used to from classical LPMO reactions with O_2_ and ascorbic acid (per second range rather than per minute range; see below). It is also worth noting that both detailed kinetic studies [12] and inferences from other studies showing reaction rates [10, 11, 50] suggest that *K*_m_ values for H_2_O_2_ are in the very low micromolar range. Reaction conditions need to be adapted accordingly; if initial H_2_O_2_ concentrations are too high, one could end up with very fast inactivation of the LPMO, perhaps even before detectable amounts of product have accumulated.

Ideally, H_2_O_2_ should be fed gradually to the reaction mixture, as shown in Fig. 4b, but this is not easy to accomplish in lab-scale reactions. Alternatively, one can regularly add small amounts of H_2_O_2_ to the reaction mixture [10, 46], which can be quite tedious and which may give a “staircase-like” LPMO activity profile since there will be an activity boost right after addition of fresh H_2_O_2_.

### Other methods for measuring LPMO activity

In 2012, Kittl et al. showed that LPMOs that are reduced in the presence of O_2_ will produce H_2_O_2_ and suggested that LPMO activity could be detected by detecting H_2_O_2_ production using the horseradish peroxidase/Amplex red assay [18]. This assay has been widely used in the field and is very handy for a quick assessment of (possible) LPMO activity, especially in cleaner samples. However, the method has multiple pitfalls, as recently discussed by Breslmayr et al. [69], and should only be used for qualitative assessments. Control reactions with free copper are advisable.

**Fig. 6:**
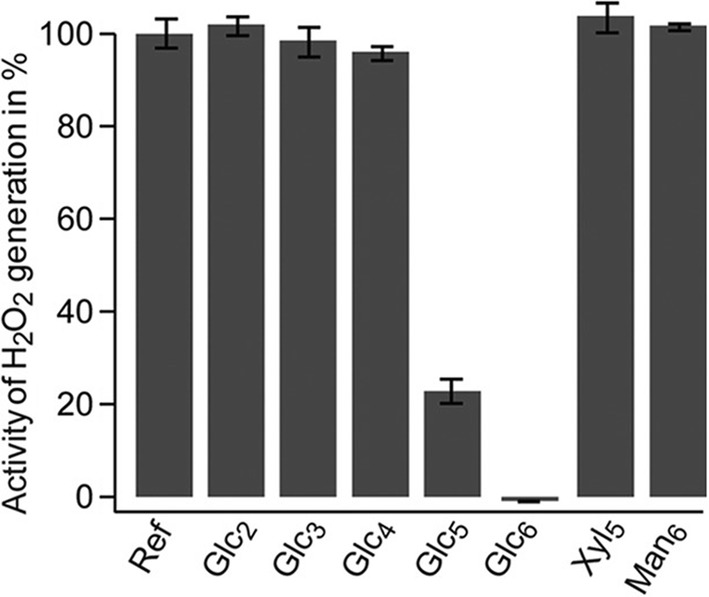
Accumulation of H_2_O_2_ when incubating *Nc*LPMO9C with reductant in the absence and presence of substrate. The enzyme (0.87 μM), which was the first LPMO for which activity on oligomeric substrates was shown, was incubated with 30 μM ascorbic acid, the reactants of the Amplex red assay and 5 mM of the indicated potential substrate, at pH 6.0 [19]. Ref, no substrate added. Control experiments without the reductant or the LPMO did not show H_2_O_2_ accumulation. Please note that the lower levels of H_2_O_2_ in reaction mixtures that contain substrates that are cleaved by the enzyme (Glc_5_ and Glc_6_) do not necessarily indicate that H_2_O_2_ was not produced, as was thought at the time; it is also possible that H_2_O_2_ was indeed produced but did not accumulate to the same extent because it was used by the LPMO when cleaving the substrate; see text for more details. This Figure was originally published in [19]

Importantly, H_2_O_2_ production is not observed if the Amplex red assay is set-up with an LPMO substrate present, and this may be very useful when screening for certain substrate specificities [19] (Fig. 6). However, in light of the recent findings concerning the ability of LPMOs to use H_2_O_2_, some of the common reasoning related to this type of experiments needs revision. The fact that H_2_O_2_ is not detected in reactions with substrate does not necessarily mean that H_2_O_2_ is not produced, as is commonly claimed; it may simply mean that produced H_2_O_2_ is consumed in productive LPMO reactions rather than for oxidation of Amplex red by horseradish peroxidase.

Frandsen et al. described an elegant method for measuring LPMO activity using derivatized cellotetraose showing FRET quenching that is relieved upon cleavage of this oligomeric substrate [23]. This is a potentially powerful and easy method which, however, for now, is only applicable for LPMOs acting on soluble substrates. Furthermore, these types of substrates are not readily available.

LPMO action reduces the molecular weight and hence leads to a decrease in the viscosity of (water-)soluble polysaccharides. Using dynamic viscosity measurements, Kojima et al. [55] were able to compare quantitatively the depolymerizing potential of two LPMOs with differing regio-specificity (the C4-oxidizing *Nc*LPMO9C from *Neurospora crassa* and the C1/C4-oxidizing *Gt*LPMO9A-2 from *Gloeophyllum trabeum*) on xyloglucan and glucomannan. It is noteworthy that dynamic viscosity measurements of LPMO activity may be more sensitive compared to HPLC and MALDI-TOF, which only detect solubilized oligosaccharides, especially when LPMO cleavage sites are located far apart on the polymeric substrate.

Vuong et al. developed an assay for measuring oxidations on the insoluble part of the substrate based on covalently linking a water-soluble fluorophore to oxidized positions within cellulose fibers [71]. When combining this analysis with standard high performance anion-exchange chromatography of soluble products, one obtains a complete picture of product formation by the LPMO. Methods for labeling C1-oxidized sites in insoluble cellulose have also be employed by Eibinger et al., who visualized adsorption of the SYTO62 fluorescent dye to carboxylic groups on the cellulose surface by confocal laser scanning microscopy [72].

Exploiting the fact that C1-oxidizing LPMOs generate carboxylic groups, Wang et al. developed an ion adsorption/desorption assay to measure oxidations on the insoluble substrate introduced by C1-oxidizing cellulose-active and chitin-active LPMOs [66]. The assay is based on incubating the insoluble reaction product (i.e., partially oxidized chitin or cellulose) with Ni^2+^, which binds to the aldonic acid groups, and spectrophotometric determination of remaining Ni^2+^ in solution using the complexometric indicator pyrocatechol violet. This method has its limitations, e.g., when it comes to quantification, but is very simple and accessible. It should be noted that the carboxylic acid product generated by a C1-oxidizing LPMO is in a pH-dependent equilibrium with its corresponding 1,5-delta lactone (alkaline pH will favor the carboxylic acid).

Interestingly, Breslmayr et al. developed a simple spectrophotometric assay that is based on the recently discovered peroxygenase activity of LPMOs, to monitor the apparent peroxidase activity of LPMOs [69]. After screening a variety of chromogenic mono-, di- and tri-phenols, 2,6-dimethoxyphenol (2,6-DMP) was selected for assay development. The LPMO oxidizes 2,6-DMP to form a radical, which dimerizes to form hydrocoerulignone, which is further oxidized by the LPMO to form coerulignone, a product with high extinction coefficient at 469 nm. While having the advantage of being simple and sensitive, this assay may suffer from interfering processes and should thus be used with care, as extensively discussed by the authors. Also, considering the fact that LPMOs show different sensitivities for inactivation by H_2_O_2_ [10, 66, 69] and likely differ in how well they interact with 2,6-DMP, the efficiency of this assay may vary between LPMOs.

### Substrate specificity

There are several ways to test the substrate specificity of LPMOs, using various natural polysaccharides, mixtures of natural polysaccharides [35, 53–55], or chromogenic substrates [51, 73]. When using non-chromogenic substrates, product formation may be assessed by MALDI-TOF MS and/or liquid chromatography. The use of MALDI-TOF MS, in principle, allows rapid screening of a wide variety of substrates. In case of complex substrates, however, the overlapping masses of various hexoses and pentoses will create problems.

Initial screening of substrate specificity entails incubating the LPMO with the to-be-tested substrates at relatively high concentration, in the presence of a reductant known to work well for LPMOs, usually ascorbic acid at a concentration in the 1 mM range. Of course, one could choose to set up reactions with H_2_O_2_ too, using for example 50 µM reductant and 100 µM H_2_O_2_. While this may seem simple, there are multiple pitfalls that need to be considered and that, in fact, make us believe that LPMOs that have been characterized so far may have activities that have been overlooked. Some pitfalls:

I. As mentioned above and discussed in more detail below, LPMOs suffer from self-inactivation (Fig. 4). The extent of this process varies between LPMOs and will be affected by the nature and concentration of the reductant and the co-substrate. Most importantly, LPMO inactivation is affected by the presence of cleavable substrate [10, 62]. It is quite possible that one sometimes “misses” certain activities because the enzyme becomes inactivated before detectable amounts of products have been produced. This may especially be true if reactants are mixed in an unfortunate order—one should avoid reduction of the LPMO in the absence of substrate.
II. As anticipated in early papers on LPMOs [51, 74], the multiplicity of these enzymes in certain biomass-degrading microorganisms suggests that some may be specialized to act on co-polymeric structures in lignocellulose, rather than on specific “pure” polysaccharides such as cellulose. Indeed, Frommhagen et al. and Couturier et al. detected LPMO activity on xylan but only when the xylan was present together with cellulose ([53, 54], respectively). In addition, we have observed that some LPMOs are able to cleave xyloglucan but only in the presence of amorphous cellulose in the reaction mixture (unpublished data). Thus, when screening the substrate specificities of LPMOs, it is advisable to also test some combinations of substrates.
III. Certain LPMO activities may not lead to soluble products and may thus be overlooked. This is underpinned by the recent discovery of a xylan-active LPMO, the founding member of the AA14 family, which acts specifically on highly refractory xylan-coated cellulose fibers [54]. The AA14s provide a spectacular example of an LPMO tailored to attack co-polymeric biomass structures that may supplement other LPMOs. Indeed, the AA14 boosted the efficiency of degradation of pretreated woody biomass by a cellulase cocktail and did so also if this cocktail was supplied with a cellulose-active LPMO. Thus, this specific AA14 activity adds efficiency to the degradation process beyond what can be achieved using cellulose-active LPMOs. While Couturier et al. initially did not detect soluble products, NMR studies indicated that the AA14 acted on xylan. Subsequent studies with added xylanases then led to the detection of oxidized xylo-oligomers. Thus, apparently, this LPMO only makes a very limited number of cuts at very specific locations that leaves the xylan chain with the oxidized end attached to cellulose. For the same reason (i.e., a limited number of cuts), Kojima et al. [55] needed to use viscosity measurements to demonstrate that an AA9 LPMO was able to depolymerize konjac glucomannan, while no oligosaccharides could be detected with HPAEC or MALDI-TOF analyses.

Another reason to sometimes use other enzymes when screening for substrate specificity may be to resolve ambiguities resulting from the fact that MS cannot discriminate between different common hexoses and pentoses. In such cases, enzymatic treatments with specific enzymes acting on only some of the possibly observed product types may be useful.

While the above addresses qualitative screening of substrate specificity, the next step in the characterization of LPMOs acting on multiple substrates would be quantitative studies of substrate preferences. While LPMOs acting on multiple substrates have been known since 2014 [19, 40, 51], to the best of our knowledge, the literature does not contain a proper comparative assessment of substrate preferences for any LPMO, apart from a few attempts [51, 55]. Such comparative studies can only be based on proper progress curves for each of the substrates and will suffer from all the complications relative to quantitative assessment of LPMO activity discussed in this review. Enzyme stability, i.e., resistance towards oxidative self-inactivation, will likely vary between substrates and one may wonder to what extent this parameter should be included when assessing the nature of the “true” substrate of an LPMO. We believe that it is well possible that non-natural reaction conditions employed in the laboratory may endorse an LPMO with activity towards substrates that are not natural substrates and that may not be biologically relevant.

### The role(s) of the reductant

From the seminal study by Kracher et al. [45] and work by others, it is clear that LPMO reactions can be fueled by a wide variety of reductants. These reductants include small molecule reductants such as ascorbic acid and several phenols [3, 5, 26, 44, 75], enzymes capable of delivering reducing equivalents, such as cellobiose dehydrogenase [15, 64, 76–79], lignin and lignin fragments [80–83], and light-driven systems [43, 84]. It is clear that the reductant (type and concentration) is a major determinant of LPMO functionality. Nice overviews of the various reducing systems may be found in [45] and [26], whereas Bissaro et al. [4] have recently reviewed the possible interplay between LPMOs and other fungal redox enzymes.

A detailed discussion of various reductants and their potential roles in LPMO catalysis is beyond the scope of this review. The role of reductants in LPMO catalysis definitively needs further attention and needs to be considered very carefully when interpreting experimental results. One of the big questions in LPMO research has sometimes been referred to as the “second electron conundrum”: if the LPMO uses O_2_ and if the LPMO has only “storage space” for one electron in the form of its single copper ion, how then is the second electron delivered to the catalytic center in the enzyme–substrate complex? Literature provides various possible answers to this question, primarily based on the existence of an electron channel (e.g., [85]) or the possibility that the LPMO recruits an electron from one of its aromatic side chains, as has been observed in other redox enzymes [86, 87]. Still, there is no consensus and LPMOs do not show conserved structural features that could be associated with any of the proposed scenarios. From the point of assessing reductant efficiency, the question is whether delivery of the first or delivery of the second electron is rate-limiting.

The discovery that H_2_O_2_ can fuel LPMO reactions potentially sheds completely new light on the role of the reductant. Indeed, assuming that H_2_O_2_ is the true co-substrate of LPMOs, the authors of this review have previously suggested that under most, if not all, conditions used so far in assessing LPMO activity, production of the co-substrate H_2_O_2_, by the LPMO and/or through direct reactions between the reductant and O_2_, is the rate-limiting factor. While this remains somewhat controversial, it is worth noting that reported rates for O_2_-driven LPMO reactions tend to be in a narrow range of 1–10 min^−1^, regardless of the type of LPMO and regardless of the substrate [4]. Some would argue that this indicates that the rate one is measuring reflects a rate-limiting process that is similar for most of these reactions, which could be production of H_2_O_2_. Loose et al. have shown that the rate of chitin oxidation by CDH-driven *Sm*LPMO10A (also known as CBP21, the family AA10 LPMO of the soil bacterium *Serratia marcescens*) is essentially identical to the rate at which CDH produces H_2_O_2_ in the presence of O_2_ as the only electron acceptor [64]. If one accepts H_2_O_2_-based catalysis, the efficiency of various reductants reflects at least in part the ability to promote production of H_2_O_2_, either directly, in solution, or in a process involving non-substrate bound LPMOs. Of note, also H_2_O_2_-based LPMO catalysis requires reduction and occasional re-reduction of the catalytic copper ion by the reductant.

It is important to note that variation in the reductant will not only affect the efficiency of the LPMO but also the occurrence of several of the side reactions listed above. Thus, the reductant will affect much more than the redox state of the LPMO, including the concentrations of O_2_ and H_2_O_2_, and the redox state of transition metals in the reaction mixture.

Very recently, using kinetics, Kuusk et al. have studied the role of the reductant in H_2_O_2_-driven degradation of chitin by *Sm*LPMO10A [13].

### Self-inactivation of LPMOs

As mentioned multiple times above, LPMOs are sensitive towards auto-catalytic oxidative inactivation, regardless of whether the reaction is driven by O_2_ or H_2_O_2_ (Fig. 4). As shown in Fig. 5, residues close to the catalytic copper, in particular the N-terminal histidine, become oxidatively damaged [10, 65]. This type of damage likely leads to copper being released in solution, although this has not yet been assessed experimentally.

Accumulating data clearly indicate that this type of damage occurs when a reduced LPMO is in solution, where it may react with O_2_ or H_2_O_2_ in the absence of a substrate, which would normally be the target for the generated powerful oxygen species. This would imply that the generated oxidative species will react on something else, such as nearby amino acid side chains on the protein, as is indeed observed. Accordingly, it has been shown that higher substrate concentrations and the presence of carbohydrate-binding modules (CBMs) improve LPMO resistance against inactivation [58, 62], whereas stability is reduced upon mutating surface residues that contribute to substrate binding [58, 65].

We suspect that the degree of auto-catalytic damage also will be affected by the type of substrate. It is clear that substrate binding helps shaping the active site of an LPMO. Substrate binding provides the confinement in the catalytic center that leads to the precise spatial orientation of the reactive oxygen species that is needed for substrate oxidation to occur and enzyme oxidation to be minimized [14, 88, 89]. Studying binding of cello- and xylo-oligomers to an LPMO by X-ray crystallography, Simmons et al. showed that these compounds, both of which are cleaved by the enzyme, bind in different ways [70]. The different binding modes result in different configurations of the catalytic centers in the enzyme–substrate complex as shown by different EPR signals indicating differences in the copper environment. Thus, different substrates may affect the reactivity of the copper site and will also affect to what extend the emerging oxidative oxygen species is confined to the one single orientation that results in productive catalysis (i.e., abstraction of a hydrogen atom from the C1 or C4 position in the substrate). For the same reasons, such variation in substrate binding may also affect the extent to which the substrate undergoes non-specific oxidations, such as those recently described in [11].

To obtain stable reactions, with no enzyme inactivation, it is thus essential to create conditions in which reduced LPMOs spend as little time in the absence of substrate as possible. Obviously, when setting up reactions, reagents need to be mixed in the right order (e.g., substrate/buffer followed by enzyme followed by at least 30 min incubation to allow the binding equilibrium to establish and finally the reductant, optionally followed by H_2_O_2_, to start the reaction) and substrate concentrations need to be as high as possible. To obtain the best possible progress curves, one may try out several reductants in various concentrations. Enzymatic electron donors such as CDH, which oxidizes cellobiose and longer cello-oligosaccharides, or the recently described pyrroloquinoline quinone-dependent (PQQ-dependent) pyranose dehydrogenase from *Coprinopsis cinerea* (*Cc*PDH), which oxidizes rare monosugars such as fucose and 2-keto–d-glucose, are less readily available but tend to yield stable kinetics in some conditions [64, 79]. Although there is no solid advice as to the optimal choice of small molecule reductants, freshly made gallic acid solutions tend to give good results in our hands. Some notes on how to best set up H_2_O_2_-driven reactions are described above.

### LPMO kinetics

Due to the many complications in assaying LPMO activity, proper kinetic data for these enzymes are scarce. In a recent review, Bissaro et al. have listed apparent LPMO rates that were published as rates or that could be deduced from published progress curves [4]. In line with the original findings of Vaaje-Kolstad et al. for the chitin-active *Sm*LPMO10A [3], published or deduced rates for O_2_-driven LPMO reactions are amazingly low, varying from 0.1 s^−1^ to below 10^−4^ s^−1^. LPMO reactions driven by H_2_O_2_ [10, 11], or by the light-chlorophyllin-reductant system [84], are much faster, with rates in the 10 s^−1^ range or even higher.

The kinetic differences between O_2_- and H_2_O_2_-driven reactions become even larger when taking into account the *K*_m_ values for the co-substrate. Studying H_2_O_2_-driven catalysis by chitin-active *Sm*LPMO10A, Kuusk et al. found a *k*_cat_ of 6.7 s^−1^ and a *K*_m_ for H_2_O_2_ of 2.8 μM. This type of values yields catalytic efficiencies (*k*_cat_/*K*_m_) in the order of 10^6^ M^−1^ s^−1^, which are values commonly observed for enzymes, including peroxygenases [12]. Kinetic studies of the O_2_-driven degradation of cellohexaose by *Mt*LPMO9E, an LPMO from the fungus *Myceliophthora thermophila*, yielded a *k*_cat_ of 0.28 s^−1^ and a *K*_m_ for O_2_ of 230 μM [11]. So, in this case, the catalytic efficiency is in the order of 10^3^ M^−1^ s^−1^, i.e., three orders of magnitude lower compared to H_2_O_2_-driven degradation of chitin.

### LPMOs in biomass conversion: some considerations

LPMOs contribute considerably to the efficiency of modern commercial cellulase cocktails used in the conversion of lignocellulosic biomass [50, 59, 60, 90–92]. The optimization of enzyme cocktails, including optimal harnessing of LPMO potential, is beyond the scope of the present paper, but it needs to be pointed out that the challenges related to LPMO research become even larger when working with true substrates. Basically, any possible side reaction listed above will occur and we suspect that enzyme inactivation is a major issue.

This complexity is well illustrated by the work of Müller et al. [50], who studied degradation of various (ligno)cellulosic substrates with Cellic CTec2 (a commercial cellulolytic enzyme cocktail produced by Novozymes) while supplying reactions with H_2_O_2_. Studies with “clean” substrates, such as Avicel, confirmed the importance of LPMOs in the enzyme cocktail, since glucan saccharification yields were more than 30% higher under conditions promoting LPMO activity. Furthermore, the use of H_2_O_2_ was favorable compared to a standard O_2_-driven reaction, giving higher LPMO activities and up to 10% higher final glucose yields. However, when using less clean, lignin-rich substrates, the situation became less clear and improvements using H_2_O_2_ were minimal. This is likely related to the fact that lignin and lignin-derived compounds may engage in various redox reactions, including reactions with H_2_O_2_.

One intriguing issue relates to the fact that LPMOs can be tuned to catalyze polysaccharide oxidation much faster than previously thought. Still, looking at the emergence of LPMO products during degradation of biomass [50] and assuming that about 15% of the protein in modern cellulolytic cocktails is LPMO ([60]; note that the number of 15% really is only an assumption with some basis in the cited study), one can deduce that the LPMOs run at rates far below 1 s^−1^. The question then is: are we actually using all the LPMO molecules in the cellulase cocktail? Or are we only using a fraction of the LPMOs, while a large majority of non-productive LPMOs slowly becomes inactivated?

Another point to consider in bioprocessing concerns the gradual depletion of substrate as the reaction proceeds. This depletion will increase the chances of LPMO inactivation, as outlined above. Indeed, the recent study by Müller et al. [50] showed that under many of the tested conditions LPMO activity ceased before the end of the reaction. It is thus conceivable that towards the end of the reaction, when possibly only the most recalcitrant part of the substrate is left and LPMO activity could be most needed, there actually is no LPMO activity left.

## Conclusion

Studying LPMO functionality is demanding. In the text above, we have addressed several complicating factors and provided some thoughts on how some of these factors could be handled. Perhaps the biggest complication lies in the nature of the co-substrate, which, in fact, is difficult to assess experimentally. The fact that the one potential co-substrate, O_2_, can be converted to another, H_2_O_2_, which leads to faster catalysis, makes experimental LPMO-work challenging.

**Fig. 7:**
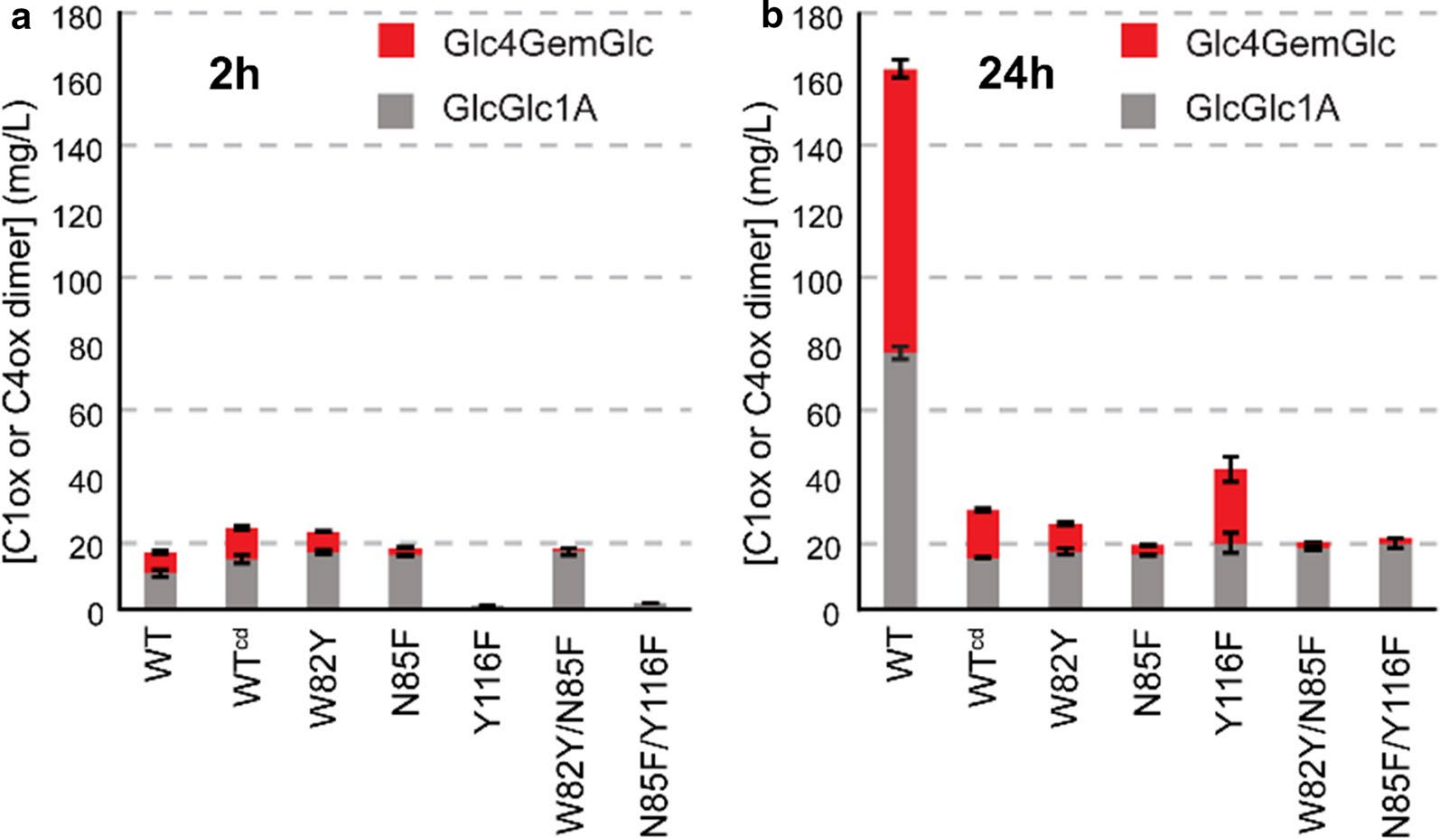
The importance of using progress curves when making quantitative statements on LPMO properties. The graphs show LPMO products generated by a series of engineered variants of a bacterial C1/C4-oxidizing LPMO from *Micromonospora aurantiaca* called *Ma*LPMO10B. **a** Product levels after 2 h; **b** product levels after 24 h. Clearly, if the mutants had been characterized by assessing only one time point, important information would have been missed and the conclusions of the study would have been strongly influenced by the choice of time point. Of note, some of the variants were likely already completely activated at 2 h (e.g., N85F), which implies that their initial catalytic rates may be higher than suggested by the product levels observed after 2 h. The data shown here are from [58]. See [65] for a similar example
- The absence of detectable H_2_O_2_ levels in reaction mixtures that contain an LPMO, a reductant and a substrate does not necessarily show that H_2_O_2_ is not produced, since produced H_2_O_2_ may be rapidly consumed by the LPMO.
- Because the substrate is very important in shaping the active site [23, 70, 89], one should be very careful when extrapolating conclusions from studies done in the absence of substrate.
- The role of the reductant may be more diverse than previously thought and could relate to its effect on production and consumption of H_2_O_2_ in the reaction mixture. The first reduction step, i.e., converting LPMO-Cu(II) to the Cu(I) form, may not be rate-limiting.
- There is no basis to claim or assume that the LPMO is principally less stable in H_2_O_2_-driven reactions compared to O_2_-driven reactions. It all just depends on getting the reaction conditions right.
- There is no basis to claim or assume that the LPMO is less specific in H_2_O_2_-driven reactions compared to O_2_-driven reactions. The occurrence of non-specific substrate oxidations likely varies between different LPMO-substrate combinations.

Based on the text above and the most recent insights into LPMO functionality, a few guidelines for future LPMO experiments seem warranted:

- It is advisable to check recombinantly produced LPMOs for an intact N-terminal histidine and to ensure copper-binding, at least if the LPMO is to be used in quantitative studies.
- Making quantitative statements about LPMO activity or substrate specificity without determining progress curves is not recommended (Fig. 7).

A final point, not addressed above, concerns the use of enzymes such as catalase or horseradish peroxidase in competition experiments set up to assess the possible role of H_2_O_2_ in LPMO catalysis (e.g., [10, 11, 88, 93]). In such experiments, it is crucial to carefully consider the competitive aspect of the experimental set up. A lack of effect of catalase or horseradish peroxidase on LPMO activity could indicate that H_2_O_2_ does not play a role in LPMO catalysis. However, an alternative explanation for such a lack of effect could be found in reaction kinetics: If the LPMO is much more efficient in utilizing emerging H_2_O_2_ than the added competitors, the latter will not inhibit the reaction (see [13] for further discussion).

There is no doubt that the LPMOs, which are remarkably abundant in Nature [54, 74, 94, 95], still hold many unanswered questions. One of the most exciting of these relates to the possible existence of other functionalities, i.e., functionalities that are not discussed above. LPMOs seem well suited to act on a wide variety of interfaces and it is likely only a matter of time before novel LPMO substrates (other polysaccharides, various recalcitrant protein fibers, lignin, or perhaps plastics) will be discovered. Another issue concerns LPMO kinetics, which remains partly unresolved. Oxygen-driven reactions tend to be exceptionally slow, whereas H_2_O_2_-driven reactions are fast, but whether O_2_ or H_2_O_2_ is the “natural” or “best” (in biorefining) co-substrate is still debated.

In the pursuit of deeper fundamental insights into LPMO enzymology a few mistakes made in the early years of LPMO research should be avoided. We hope that this paper contributes to increasing the quality of future LPMO research by ourselves and others and that these fascinating enzymes will continue to excite and surprise us.

## Abbreviations

LPMO: lytic polysaccharide monooxygenase
CDH: cellobiose dehydrogenase
PDH: pyranose dehydrogenase
TXG: tamarind xyloglucan
HILIC: hydrophilic interaction chromatography
HPAEC-PAD: high performance anion exchange chromatography with pulsed amperometric detection
AA: auxiliary activity
FRET: fluorescence resonance energy transfer
MALDI-TOF MS: matrix-assisted laser desorption ionization–time of flight mass spectrometry
EPR: electron paramagnetic resonance

## References

[1] Vaaje-Kolstad G, Horn SJ, van Aalten DMF, Synstad B, Eijsink VGH (2005). The non-catalytic chitin-binding protein CBP21 from *Serratia marcescens* is essential for chitin degradation. J Biol Chem.

[2] Merino ST, Cherry J (2007). Progress and challenges in enzyme development for Biomass utilization. Biofuels..

[3] Vaaje-Kolstad G, Westereng B, Horn SJ, Liu ZL, Zhai H, Sørlie M, Eijsink VGH (2010). An oxidative enzyme boosting the enzymatic conversion of recalcitrant polysaccharides. Science.

[4] Bissaro B, Varnai A, Røhr AK, Eijsink VGH (2018). Oxidoreductases and reactive oxygen species in conversion of lignocellulosic biomass. Microbiol Mol Biol R..

[5] Quinlan RJ, Sweeney MD, Lo Leggio L, Otten H, Poulsen JC, Johansen KS, Krogh KB, Jorgensen CI, Tovborg M, Anthonsen A, Tryfona T, Walter CP, Dupree P, Xu F, Davies GJ, Walton PH (2011). Insights into the oxidative degradation of cellulose by a copper metalloenzyme that exploits biomass components. Proc Natl Acad Sci USA.

[6] Aachmann FL, Sørlie M, Skjåk-Bræk G, Eijsink VGH, Vaaje-Kolstad G (2012). NMR structure of a lytic polysaccharide monooxygenase provides insight into copper-binding, protein dynamics, and substrate interactions. Proc Natl Acad Sci USA.

[7] Hemsworth GR, Taylor EJ, Kim RQ, Gregory RC, Lewis SJ, Turkenburg JP, Parkin A, Davies GJ, Walton PH (2013). The copper active site of CBM33 polysaccharide oxygenases. J Am Chem Soc.

[8] Ciano L, Davies GJ, Tolman WB, Walton PH (2018). Bracing copper for the catalytic oxidation of C–H bonds. Nat Catal..

[9] Beeson WT, Phillips CM, Cate JH, Marletta MA (2012). Oxidative cleavage of cellulose by fungal copper-dependent polysaccharide monooxygenases. J Am Chem Soc.

[10] Bissaro B, Røhr AK, Müller G, Chylenski P, Skaugen M, Forsberg Z, Horn SJ, Vaaje-Kolstad G, Eijsink VGH (2017). Oxidative cleavage of polysaccharides by monocopper enzymes depends on H_2_O_2_. Nat Chem Biol.

[11] Hangasky JA, Iavarone AT, Marletta MA (2018). Reactivity of O_2_ versus H_2_O_2_ with polysaccharide monooxygenases. Proc Natl Acad Sci USA.

[12] Kuusk S, Bissaro B, Kuusk P, Forsberg Z, Eijsink VGH, Sørlie M, Valjamae P (2018). Kinetics of H_2_O_2_-driven degradation of chitin by a bacterial lytic polysaccharide monooxygenase. J Biol Chem.

[13] Kuusk S, Kont R, Kuusk P, Heering A, Sørlie M, Bissaro B, Eijsink VGH, Valjamae P (2019). Kinetic insights into the role of the reductant in H_2_O_2_-driven degradation of chitin by a bacterial lytic polysaccharide monooxygenase. J Biol Chem.

[14] Wang BJ, Johnston EM, Li PF, Shaik S, Davies GJ, Walton PH, Rovira C (2018). QM/MM studies into the H_2_O_2_-dependent activity of lytic polysaccharide monooxygenases: evidence for the formation of a caged hydroxyl radical intermediate. ACS Catal..

[15] Phillips CM, Beeson WT, Cate JH, Marletta MA (2011). Cellobiose dehydrogenase and a copper-dependent polysaccharide monooxygenase potentiate cellulose degradation by *Neurospora crassa*. ACS Chem Biol.

[16] Beeson WT, Vu VV, Span EA, Phillips CM, Marletta MA (2015). Cellulose degradation by polysaccharide monooxygenases. Annu Rev Biochem.

[17] Walton PH, Davies GJ (2016). On the catalytic mechanisms of lytic polysaccharide monooxygenases. Curr Opin Chem Biol.

[18] Kittl R, Kracher D, Burgstaller D, Haltrich D, Ludwig R (2012). Production of four *Neurospora crassa* lytic polysaccharide monooxygenases in *Pichia pastoris* monitored by a fluorimetric assay. Biotechnol Biofuels.

[19] Isaksen T, Westereng B, Aachmann FL, Agger JW, Kracher D, Kittl R, Ludwig R, Haltrich D, Eijsink VGH, Horn SJ (2014). A C4-oxidizing lytic polysaccharide monooxygenase cleaving both cellulose and cello-oligosaccharides. J Biol Chem.

[20] Lombard V, Golaconda Ramulu H, Drula E, Coutinho PM, Henrissat B (2014). The carbohydrate-active enzymes database (CAZy) in 2013. Nucleic Acids Res.

[21] Levasseur A, Drula E, Lombard V, Coutinho PM, Henrissat B (2013). Expansion of the enzymatic repertoire of the CAZy database to integrate auxiliary redox enzymes. Biotechnol Biofuels.

[22] Westereng B, Agger JW, Horn SJ, Vaaje-Kolstad G, Aachmann FL, Stenstrøm YH, Eijsink VGH (2013). Efficient separation of oxidized cello-oligosaccharides generated by cellulose degrading lytic polysaccharide monooxygenases. J Chromatogr A.

[23] Frandsen KE, Simmons TJ, Dupree P, Poulsen JC, Hemsworth GR, Ciano L, Johnston EM, Tovborg M, Johansen KS, von Freiesleben P, Marmuse L, Fort S, Cottaz S, Driguez H, Henrissat B, Lenfant N, Tuna F, Baldansuren A, Davies GJ (2016). The molecular basis of polysaccharide cleavage by lytic polysaccharide monooxygenases. Nat Chem Biol.

[24] Westereng B, Arntzen MO, Aachmann FL, Varnai A, Eijsink VGH, Agger JW (2016). Simultaneous analysis of C1 and C4 oxidized oligosaccharides, the products of lytic polysaccharide monooxygenases acting on cellulose. J Chromatogr A.

[25] Westereng B, Arntzen MO, Agger JW, Vaaje-Kolstad G, Eijsink VGH (2017). Analyzing activities of lytic polysaccharide monooxygenases by liquid chromatography and mass spectrometry. Methods Mol Biol.

[26] Frommhagen M, Westphal AH, van Berkel WJH, Kabel MA (2018). Distinct substrate specificities and electron-donating systems of fungal lytic polysaccharide monooxygenases. Front Microbiol..

[27] Hemsworth GR, Ciano L, Davies GJ, Walton PH (2018). Production and spectroscopic characterization of lytic polysaccharide monooxygenases. Methods Enzymol.

[28] Westereng B, Loose JSM, Vaaje-Kolstad G, Aachmann FL, Sørlie M, Eijsink VGH (2018). Analytical tools for characterizing cellulose-active lytic polysaccharide monooxygenases (LPMOs). Methods Mol Biol.

[29] Kadowaki MAS, Varnai A, Jameson JK, Leite AET, Costa AJ, Kumagai PS, Prade RA, Polikarpov I, Eijsink VGH (2018). Functional characterization of a lytic polysaccharide monooxygenase from the thermophilic fungus *Myceliophthora thermophila*. PLoS ONE.

[30] Ong E, Kilburn DG, Miller RC, Warren RA (1994). *Streptomyces lividans* glycosylates the linker region of a beta-1,4-glycanase from *Cellulomonas fimi*. J Bacteriol.

[31] Poon DK, Withers SG, McIntosh LP (2007). Direct demonstration of the flexibility of the glycosylated proline-threonine linker in the *Cellulomonas fimi* Xylanase Cex through NMR spectroscopic analysis. J Biol Chem.

[32] Borisova AS, Isaksen T, Dimarogona M, Kognole AA, Mathiesen G, Varnai A, Rohr AK, Payne CM, Sorlie M, Sandgren M, Eijsink VG (2015). Structural and functional characterization of a lytic polysaccharide monooxygenase with broad substrate specificity. J Biol Chem.

[33] Liu B, Kognole AA, Wu M, Westereng B, Crowley MF, Kim S, Dimarogona M, Payne CM, Sandgren M (2018). Structural and molecular dynamics studies of a C1-oxidizing lytic polysaccharide monooxygenase from *Heterobasidion irregulare* reveal amino acids important for substrate recognition. FEBS J.

[34] Wu M, Beckham GT, Larsson AM, Ishida T, Kim S, Payne CM, Himmel ME, Crowley MF, Horn SJ, Westereng B, Igarashi K, Samejima M, Stahlberg J, Eijsink VGH, Sandgren M (2013). Crystal structure and computational characterization of the lytic polysaccharide monooxygenase GH61D from the Basidiomycota fungus *Phanerochaete chrysosporium*. J Biol Chem.

[35] Petrovic DM, Bissaro B, Chylenski P, Skaugen M, Sørlie M, Jensen MS, Aachmann FL, Courtade G, Varnai A, Eijsink VGH (2018). Methylation of the N-terminal histidine protects a lytic polysaccharide monooxygenase from auto-oxidative inactivation. Protein Sci.

[36] Crouch LI, Labourel A, Walton PH, Davies GJ, Gilbert HJ (2016). The contribution of non-catalytic carbohydrate binding modules to the activity of lytic polysaccharide monooxygenases. J Biol Chem.

[37] Gardner JG, Crouch L, Labourel A, Forsberg Z, Bukhman YV, Vaaje-Kolstad G, Gilbert HJ, Keating DH (2014). Systems biology defines the biological significance of redox-active proteins during cellulose degradation in an aerobic bacterium. Mol Microbiol.

[38] Chaplin AK, Wilson MT, Hough MA, Svistunenko DA, Hemsworth GR, Walton PH, Vijgenboom E, Worrall JA (2016). Heterogeneity in the histidine-brace copper coordination sphere in auxiliary activity family 10 (AA10) lytic polysaccharide monooxygenases. J Biol Chem.

[39] Loose JS, Forsberg Z, Fraaije MW, Eijsink VGH, Vaaje-Kolstad G (2014). A rapid quantitative activity assay shows that the *Vibrio cholerae* colonization factor GbpA is an active lytic polysaccharide monooxygenase. FEBS Lett.

[40] Forsberg Z, Mackenzie AK, Sørlie M, Røhr AK, Helland R, Arvai AS, Vaaje-Kolstad G, Eijsink VGH (2014). Structural and functional characterization of a conserved pair of bacterial cellulose-oxidizing lytic polysaccharide monooxygenases. Proc Natl Acad Sci USA.

[41] Xiao Z, Wedd AG (2010). The challenges of determining metal-protein affinities. Nat Prod Rep.

[42] Gregory RC, Hemsworth GR, Turkenburg JP, Hart SJ, Walton PH, Davies GJ (2016). Activity, stability and 3-D structure of the Cu(II) form of a chitin-active lytic polysaccharide monooxygenase from *Bacillus amyloliquefaciens*. Dalton Trans.

[43] Bissaro B, Forsberg Z, Ni Y, Hollmann F, Vaaje-Kolstad G, Eijsink VGH (2016). Fueling biomass-degrading oxidative enzymes by light-driven water oxidation. Green Chem.

[44] Frommhagen M, Koetsier MJ, Westphal AH, Visser J, Hinz SW, Vincken JP, van Berkel WJ, Kabel MA, Gruppen H (2016). Lytic polysaccharide monooxygenases from *Myceliophthora thermophila* C1 differ in substrate preference and reducing agent specificity. Biotechnol Biofuels.

[45] Kracher D, Scheiblbrandner S, Felice AK, Breslmayr E, Preims M, Ludwicka K, Haltrich D, Eijsink VGH, Ludwig R (2016). Extracellular electron transfer systems fuel cellulose oxidative degradation. Science.

[46] Hegnar OA, Petrovic DM, Bissaro B, Alfredsen G, Varnai A, Eijsink VGH (2019). pH-dependent relationship between catalytic activity and hydrogen peroxide production shown via characterization of a lytic polysaccharide monooxygenase from *Gloeophyllum trabeum*. Appl Environ Microbiol.

[47] Wood TM (1988). Preparation of crystalline, amorphous, and dyed cellulase substrates. Method Enzymol..

[48] Cuong HN, Minh NC, Van Hoa N, Trung TS (2016). Preparation and characterization of high purity beta-chitin from squid pens (*Loligo chenisis*). Int J Biol Macromol.

[49] Vu VV, Beeson WT, Phillips CM, Cate JH, Marletta MA (2014). Determinants of regioselective hydroxylation in the fungal polysaccharide monooxygenases. J Am Chem Soc.

[50] Müller G, Chylenski P, Bissaro B, Eijsink VGH, Horn SJ (2018). The impact of hydrogen peroxide supply on LPMO activity and overall saccharification efficiency of a commercial cellulase cocktail. Biotechnol Biofuels.

[51] Agger JW, Isaksen T, Varnai A, Vidal-Melgosa S, Willats WG, Ludwig R, Horn SJ, Eijsink VGH, Westereng B (2014). Discovery of LPMO activity on hemicelluloses shows the importance of oxidative processes in plant cell wall degradation. Proc Natl Acad Sci USA.

[52] Bennati-Granier C, Garajova S, Champion C, Grisel S, Haon M, Zhou S, Fanuel M, Ropartz D, Rogniaux H, Gimbert I, Record E, Berrin JG (2015). Substrate specificity and regioselectivity of fungal AA9 lytic polysaccharide monooxygenases secreted by *Podospora anserina*. Biotechnol Biofuels.

[53] Frommhagen M, Sforza S, Westphal AH, Visser J, Hinz SW, Koetsier MJ, van Berkel WJ, Gruppen H, Kabel MA (2015). Discovery of the combined oxidative cleavage of plant xylan and cellulose by a new fungal polysaccharide monooxygenase. Biotechnol Biofuels.

[54] Couturier M, Ladeveze S, Sulzenbacher G, Ciano L, Fanuel M, Moreau C, Villares A, Cathala B, Chaspoul F, Frandsen KE, Labourel A, Herpoel-Gimbert I, Grisel S, Haon M, Lenfant N, Rogniaux H, Ropartz D, Davies GJ, Rosso MN (2018). Lytic xylan oxidases from wood-decay fungi unlock biomass degradation. Nat Chem Biol.

[55] Kojima Y, Varnai A, Ishida T, Sunagawa N, Petrovic DM, Igarashi K, Jellison J, Goodell B, Alfredsen G, Westereng B, Eijsink VGH, Yoshida M (2016). A lytic polysaccharide monooxygenase with broad xyloglucan specificity from the brown-rot fungus *Gloeophyllum trabeum* and its action on cellulose-xyloglucan complexes. Appl Environ Microbiol.

[56] Bao WJ, Usha SN, Renganathan V (1993). Purification and characterization of cellobiose dehydrogenase, a novel extracellular hemoflavoenzyme from the white-rot fungus *Phanerochaete chrysosporium*. Arch Biochem Biophys.

[57] Harreither W, Coman V, Ludwig R, Haltrich D, Gorton L (2007). Investigation of graphite electrodes modified with cellobiose dehydrogenase from the ascomycete *Myriococcum thermophilum*. Electroanal..

[58] Forsberg Z, Bissaro B, Gullesen J, Dalhus B, Vaaje-Kolstad G, Eijsink VGH (2018). Structural determinants of bacterial lytic polysaccharide monooxygenase functionality. J Biol Chem.

[59] Cannella D, Hsieh CW, Felby C, Jorgensen H (2012). Production and effect of aldonic acids during enzymatic hydrolysis of lignocellulose at high dry matter content. Biotechnol Biofuels.

[60] Müller G, Varnai A, Johansen KS, Eijsink VGH, Horn SJ (2015). Harnessing the potential of LPMO-containing cellulase cocktails poses new demands on processing conditions. Biotechnol Biofuels.

[61] Heuts DP, Winter RT, Damsma GE, Janssen DB, Fraaije MW (2008). The role of double covalent flavin binding in chito-oligosaccharide oxidase from *Fusarium graminearum*. Biochem J..

[62] Courtade G, Forsberg Z, Heggset EB, Eijsink VGH, Aachmann FL (2018). The carbohydrate-binding module and linker of a modular lytic polysaccharide monooxygenase promote localized cellulose oxidation. J Biol Chem.

[63] Frommhagen M, Westphal AH, Hilgers R, Koetsier MJ, Hinz SWA, Visser J, Gruppen H, van Berkel WJH, Kabel MA (2018). Quantification of the catalytic performance of C1-cellulose-specific lytic polysaccharide monooxygenases. Appl Microbiol Biot..

[64] Loose JS, Forsberg Z, Kracher D, Scheiblbrandner S, Ludwig R, Eijsink VGH, Vaaje-Kolstad G (2016). Activation of bacterial lytic polysaccharide monooxygenases with cellobiose dehydrogenase. Protein Sci.

[65] Loose JSM, Arntzen MO, Bissaro B, Ludwig R, Eijsink VGH, Vaaje-Kolstad G (2018). Multipoint precision binding of substrate protects lytic polysaccharide monooxygenases from self-destructive off-pathway processes. Biochemistry.

[66] Wang DM, Li J, Wong ACY, Aachmann FL, Hsieh YSY (2018). A colorimetric assay to rapidly determine the activities of lytic polysaccharide monooxygenases. Biotechnol Biofuels.

[67] Scott BR, Huang HZ, Frickman J, Halvorsen R, Johansen KS (2016). Catalase improves saccharification of lignocellulose by reducing lytic polysaccharide monooxygenase-associated enzyme inactivation. Biotechnol Lett.

[68] Weiss RF (1970). Solubility of nitrogen, oxygen and argon in water and seawater. Deep-Sea Res.

[69] Breslmayr E, Hanzek M, Hanrahan A, Leitner C, Kittl R, Santek B, Oostenbrink C, Ludwig R (2018). A fast and sensitive activity assay for lytic polysaccharide monooxygenase. Biotechnol Biofuels.

[70] Simmons TJ, Frandsen KEH, Ciano L, Tryfona T, Lenfant N, Poulsen JC, Wilson LFL, Tandrup T, Tovborg M, Schnorr K, Johansen KS, Henrissat B, Walton PH, Lo Leggio L, Dupree P (2017). Structural and electronic determinants of lytic polysaccharide monooxygenase reactivity on polysaccharide substrates. Nat Commun..

[71] Vuong TV, Liu B, Sandgren M, Master ER (2017). Microplate-based detection of lytic polysaccharide monooxygenase activity by fluorescence-labeling of insoluble oxidized products. Biomacromol.

[72] Eibinger M, Ganner T, Bubner P, Rosker S, Kracher D, Haltrich D, Ludwig R, Plank H, Nidetzky B (2014). Cellulose surface degradation by a lytic polysaccharide monooxygenase and its effect on cellulase hydrolytic efficiency. J Biol Chem.

[73] Vidal-Melgosa S, Pedersen HL, Schuckel J, Arnal G, Dumon C, Amby DB, Monrad RN, Westereng B, Willats WG (2015). A new versatile microarray-based method for high throughput screening of carbohydrate-active enzymes. J Biol Chem.

[74] Horn SJ, Vaaje-Kolstad G, Westereng B, Eijsink VGH (2012). Novel enzymes for the degradation of cellulose. Biotechnol Biofuels.

[75] Frommhagen M, Mutte SK, Westphal AH, Koetsier MJ, Hinz SWA, Visser J, Vincken JP, Weijers D, van Berkel WJH, Gruppen H, Kabel MA (2017). Boosting LPMO-driven lignocellulose degradation by polyphenol oxidase-activated lignin building blocks. Biotechnol Biofuels.

[76] Langston JA, Shaghasi T, Abbate E, Xu F, Vlasenko E, Sweeney MD (2011). Oxidoreductive cellulose depolymerization by the enzymes cellobiose dehydrogenase and glycoside hydrolase 61. Appl Environ Microbiol.

[77] Tan TC, Kracher D, Gandini R, Sygmund C, Kittl R, Haltrich D, Hallberg BM, Ludwig R, Divne C (2015). Structural basis for cellobiose dehydrogenase action during oxidative cellulose degradation. Nat Commun..

[78] Garajova S, Mathieu Y, Beccia MR, Bennati-Granier C, Biaso F, Fanuel M, Ropartz D, Guigliarelli B, Record E, Rogniaux H, Henrissat B, Berrin JG (2016). Single-domain flavoenzymes trigger lytic polysaccharide monooxygenases for oxidative degradation of cellulose. Sci Rep..

[79] Varnai A, Umezawa K, Yoshida M, Eijsink VGH (2018). The pyrroloquinoline-quinone-dependent pyranose dehydrogenase from *Coprinopsis cinerea* drives lytic polysaccharide monooxygenase action. Appl Environ Microbiol.

[80] Hu JG, Arantes V, Pribowo A, Gourlay K, Saddler JN (2014). Substrate factors that influence the synergistic interaction of AA9 and cellulases during the enzymatic hydrolysis of biomass. Energy Environ Sci..

[81] Westereng B, Cannella D, Wittrup Agger J, Jorgensen H, Larsen Andersen M, Eijsink VGH, Felby C (2015). Enzymatic cellulose oxidation is linked to lignin by long-range electron transfer. Sci Rep..

[82] Brenelli L, Squina FM, Felby C, Cannella D (2018). Laccase-derived lignin compounds boost cellulose oxidative enzymes AA9. Biotechnol Biofuels.

[83] Muraleedharan MN, Zouraris D, Karantonis A, Topakas E, Sandgren M, Rova U, Christakopoulos P, Karnaouri A (2018). Effect of lignin fractions isolated from different biomass sources on cellulose oxidation by fungal lytic polysaccharide monooxygenases. Biotechnol Biofuels.

[84] Cannella D, Mollers KB, Frigaard NU, Jensen PE, Bjerrum MJ, Johansen KS, Felby C (2016). Light-driven oxidation of polysaccharides by photosynthetic pigments and a metalloenzyme. Nat Commun..

[85] Li X, Beeson WT, Phillips CM, Marletta MA, Cate JH (2012). Structural basis for substrate targeting and catalysis by fungal polysaccharide monooxygenases. Structure..

[86] Whittaker JW (2003). Free radical catalysis by galactose oxidase. Chem Rev.

[87] Nordlund P, Reichard P (2006). Ribonucleotide reductases. Annu Rev Biochem.

[88] Bissaro B, Røhr AK, Skaugen M, Forberg Z, Horn SJ, Vaaje-Kolstad G, Eijsink VGH (2017). Fenton-type chemistry by a copper enzyme: molecular mechanism of polysaccharide oxidative cleavage. bioRxiv.

[89] Bissaro B, Isaksen I, Vaaje-Kolstad G, Eijsink VGH, Røhr AK (2018). How a lytic polysaccharide monooxygenase binds crystalline chitin. Biochemistry.

[90] Harris PV, Xu F, Kreel NE, Kang C, Fukuyama S (2014). New enzyme insights drive advances in commercial ethanol production. Curr Opin Chem Biol.

[91] Hu J, Chandra R, Arantes V, Gourlay K, Susan van Dyk J, Saddler JN (2015). The addition of accessory enzymes enhances the hydrolytic performance of cellulase enzymes at high solid loadings. Biores Tech..

[92] Johansen KS (2016). Discovery and industrial applications of lytic polysaccharide mono-oxygenases. Biochem Soc Trans.

[93] Mollers KB, Mikkelsen H, Simonsen TI, Cannella D, Johansen KS, Bjerrum MJ, Felby C (2017). On the formation and role of reactive oxygen species in light-driven LPMO oxidation of phosphoric acid-swollen cellulose. Carbohydr Res.

[94] Lenfant N, Hainaut M, Terrapon N, Drula E, Lombard V, Henrissat B (2017). A bioinformatics analysis of 3400 lytic polysaccharide oxidases from family AA9. Carbohydr Res.

[95] Sabbadin F, Hemsworth GR, Ciano L, Henrissat B, Dupree P, Tryfona T, Marques RDS, Sweeney ST, Besser K, Elias L, Pesante G, Li Y, Dowle AA, Bates R, Gomez LD, Simister R, Davies GJ, Walton PH, Bruce NC (2018). An ancient family of lytic polysaccharide monooxygenases with roles in arthropod development and biomass digestion. Nat Commun..

